# Design and Testing of Inertial System for Landslide Displacement Distribution Measurement

**DOI:** 10.3390/s20247154

**Published:** 2020-12-14

**Authors:** Yongquan Zhang, Huiming Tang, Guiying Lu, Yuansheng Wang, Changdong Li, Junrong Zhang, Pengju An, Peiwu Shen

**Affiliations:** 1 Faculty of Engineering, China University of Geosciences, Wuhan 430074, China; zhangyq@cug.edu.cn (Y.Z.); lichangdong@cug.edu.cn (C.L.); zjr@cug.edu.cn (J.Z.); apj@cug.edu.cn (P.A.); pwshen@cug.edu.cn (P.S.); 2 School of Mechanical Engineering and Electronic Information, China University of Geosciences, Wuhan 430074, China; wangyuansheng@cug.edu.cn

**Keywords:** landslide, displacement monitoring, displacement distribution measurement, inertial system, pipeline trajectory measurement

## Abstract

Landslide displacement monitoring plays a fundamental role in the study of landslide evolution mechanisms, forecasting, risk assessment, prevention, and control. To fill the deficiencies of traditional instrumentation for measuring landslide displacement distributed along lateral direction, a landslide displacement measurement method based on deformation-coupled pipeline trajectory measurement is proposed, and a pipeline trajectory inertial measurement instrument is developed. The developed instrument, primarily comprised of a single shaft gyro, two axis accelerometers, and an external roller encoder, is designed as an axial half strapdown-radial half platform structure combined with a mechanical gravity platform. This structure avoids the singularity of pitch angle and roll angle and can expediently calculate a pipeline trajectory with an Eulerian transformation when obtaining several basic physical variables, e.g., the axial linear velocity, pitch angle, roll angle, and azimuth angle. Additionally, the pipeline trajectory, measured at different times, possesses the ability to reflect the displacement evolution feature of landslides. The results of prototype simulation tests imply a single measurement accuracy of a 12 cm/100 m span and a singly periodic multiple (more than five times) measurement accuracy of a 3 cm/100 m span, which meets medium-precision displacement measurement requirements for a landslide. Additionally, the finished instrument has been successfully applied to the deformation monitoring of the Majiagou I# landslide, which further verifies its feasibility and offers a reference for similar landslides.

## 1. Introduction

Landslides are widely distributed all over the world [1,2,3,4]. It is of great significance to perform displacement monitoring of landslides for the purpose of risk assessment and engineering prevention [5]. Many researchers have extensively studied the monitoring methods and techniques of displacement regarding the slope surface and underground detection [6,7,8,9].

Displacement monitoring of the slope surface is usually performed on a regional scale of a few kilometers. Techniques such as Aautomated Total Stations monitoring, Global Positioning System (GPS), Synthetic Aperture Radar (InSAR), and Light Detection and Ranging (LiDAR) are commonly applied due to their high accuracy [10,11,12,13]. However, these monitoring methods are easily affected by terrain and other features (such as vegetation and human activities). Underground displacement monitoring is appropriate for a single landslide. This approach is able to obtain local deformation over an entire landslide mass from instruments arranged along vertical boreholes. The common methods for underground displacement monitoring include borehole inclinometers [14,15], fiber Bragg gratings (FBGs) [16,17], and time-domain reflection coaxial cables (TDRs) [18]. Since underground monitoring has an advantage in tracking the internal deformation of a landslide, it is more effective for predicting the trend of a creep landslide than surface monitoring. However, these underground monitoring instruments are vulnerable, particularly under large deformation conditions.

Considering the perspective of measurement principles, the monitoring techniques for underground displacements can be classified into two types: probe strain-based measurements and landslide attitude-based measurements. The former requires probes attached to vertical structures to measure the strain. The instruments used in this technique are vulnerable due to the large deformations that are in sync with the structures. The common instruments for this technique include FBGs and TDRs [19,20], whose deformation measuring ranges are limited. The typical instrument used for attitude-based measurement is an inclinometer, which acquires the displacement by measuring the attitude angle of scattered points in the borehole and is not dependent on the deformation of the instrument itself. The use of inclinometers is a widely accepted monitoring technique due to their large range and high accuracy [14,21].

A borehole inclinometer based on accelerometers is a simplified inertial measurement instrument that has been widely applied in the monitoring of deep displacement of landslides [15,22,23,24]. However, inclinometers are simply assembled in vertical boreholes due to the limitations of the measurement mechanism and instrument structure [24]. Referring to a deep displacement inclinometer and based on the technique of inertial measurement [25], we develop a new instrument and method for monitoring landslide displacement distribution along a horizontal direction. The procedure of this method is as follows: First, a flexible pipe is buried throughout the landslide body to deform with the landslide synchronously. Then, the developed instrument runs through the pipe to collect the path data at regular intervals. Finally, the differences in pipe paths among these intervals are calculated to analyze the displacement of the landslide.

## 2. Introduction of Inertial Measurement in Landslide Displacement Measurement

Most measurement conditions with regard to the deep displacement distribution of landslides are placed in an enclosed underground space, specifically, at the interior of landslides. However, these conditions enhance the complexity of traditional measurement methods, e.g., methods related to light, magnetism, and sound. Herein, an inertial system possessing autonomous enclosure and extensive adaptability is especially propitious to the deep displacement distribution measurement of landslides.

### 2.1. Matching of Inertial System

When referring to the inertial measurement for the deep displacement distribution of a landslide body, the intuitive impression is that the landslide body should be considered an inertial carrier, and its motion state should be effectively determined based on acceleration and angular velocity as monitored by an inertial measurement unit [26]. This measurement method, however, has been disproved in consideration of the analytical results of spatial-temporal features and inertial measurement principles related to the deformation of landslide bodies.

Generally, three forms can be concluded to describe the motion state of landslides, namely, creep landslides, progressive failure landslides and high-speed landslides. Sequentially, a creep landslide is a landslide suffering slow deformation, and a progressive failure landslide describes a landslide possessing three deformation stages: an initial deformation, a uniform deformation and an accelerating deformation [3,27]. However, a high-speed failure landslide is a large deformation landslide occurring within a short time. Due to the limitations of monitoring technology at present, the deformation of landslides is monitored for a long time, e.g., from the identification to the failure of a landslide [28]. Nevertheless, the deformation always stays at a creep state with a velocity of cm/year for general landslides, and the differential result implies that the acceleration is so small that the variation cannot be monitored.

Thus, the deep displacement distribution of landslide bodies cannot be directly measured by inertial systems due to the nonnegligible shortage that the landslide body is quasistatic relative to inertial systems, which leads to oblivion of dynamic-loading inertial parameters according to the long-term integral principle of inertial measurement. Consequentially, it is necessary to conduct research on the measurement of the deep displacement distribution of landslides based on direct measurement methods or short-term inertial parameters.

### 2.2. Establishment of Measurement Method

**Figure 1:**
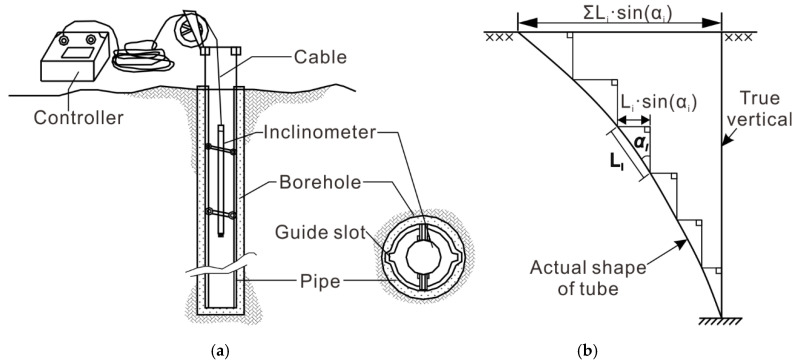
Displacement vertical distribution measurement by borehole clinometer: (**a**) Schematic diagram of borehole clinometer; (**b**) schematic diagram of cumulative displacement curves [15].

The traditional borehole clinometer for landslides is a simple inertial measurement tool and merely contains one vertical measurement line, which can be referenced to build an effective space-time conversion mode (Figure 1) [15]. The inclinometer is vertically installed in the landslide with the bottom embedded in a stable sliding bed. This placement can ensure that part of the inclinometer in the landslide body exhibits coordination deformation, and the rest of the inclinometer in the sliding bed remains stationary, offering a reference position for the deformation. Thus, the accumulation over time of acceleration and velocity of the landslide body is transformed into the absolute displacement in space. Herein, the displacement of the landslide body can be confirmed through borehole attitude measurements with an inclinometer over time, which only requires short-term measurements at a time.

**Figure 2:**
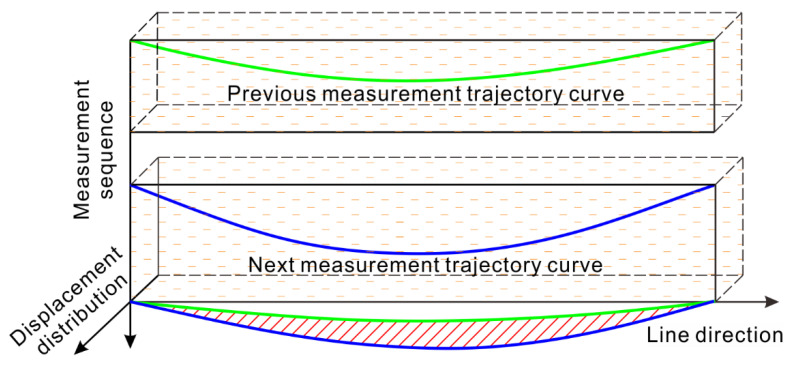
Schematic diagram of the change in the buried pipeline trajectory reflecting the displacement distribution of the landslide body.

Learning from the principle of the borehole clinometer, a pipeline, used for coupling the deformation of a landslide body, is arranged in the landslide body along the direction of the displacement distribution to be measured. Then, the trajectory of the pipeline is measured over time, and the difference in the trajectory shapes is considered to be the displacement of the landslide within the interval between two samples, as shown in Figure 2. Therefore, the next key task is to design an instrument that can acquire the pipeline trajectory [29,30,31].

## 3. Measuring Instrument Design

### 3.1. Overall Design Scheme

Assuming that the trajectory curve of the deformed coupled pipe is S, then the displacement of the landslide body is the difference in the pipeline trajectory measured at different times, that is:

(1) $$D=S_{n}-S_{0}$$

where $S_{n}$ is the pipe trajectory curve measured at the nth time; $S_{0}$ is the reference trajectory curve of the pipe, which is generally the initial trajectory when the pipe is buried.

To obtain a three-dimensional trajectory of the pipeline, the linear motion on the y-axis and the angular motion of the 3 axes must be measured; the selectable physical quantities are shown in Table 1. According to the landslide site measurement conditions, the angle around the x-axis and the y-axis can be directly obtained by reference to the gravitational g, so the parameter combination is selected as the y-axis velocity, the angle around the x-axis (defined as pitch angle θ), the angle around the y-axis (defined as roll angle γ), and the angular velocity around the z-axis (defined as yaw velocity $\omega_{z}$).

**Figure 3:**
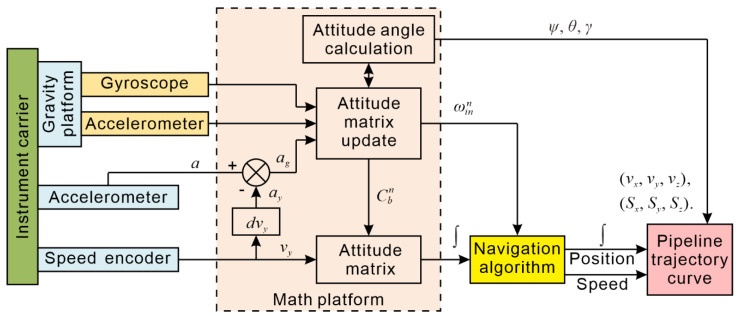
Schematic diagram of the pipeline trajectory inertial measurement system.

Based on the coupling pipe condition, by selecting the combination of measurement parameters marked by “√” in Table 1, the overall instrument design scheme shown in Figure 3 is constructed. The measuring system is a semiplatform and semistrap combined measurement system, with the y-axis for the platform and the x-axis and z-axis for the strap-down. The measurement components required by the system include one gyroscope, two gravity accelerometers and one encoder, thus maximizing hardware cost savings.

### 3.2. Mechanical Structure

**Figure 4:**
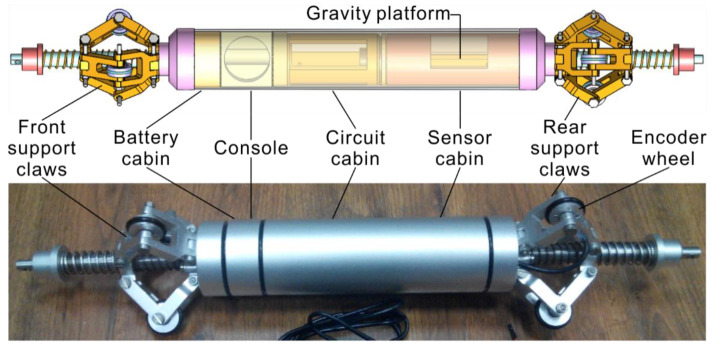
Mechanical structure of the pipeline track measurement system.

The mechanical structure of the pipeline track measurement system is shown in Figure 4 and includes six parts: the front support claw, battery cabin, console, circuit cabin, sensor cabin and rear support claw.

The overall structure of the instrument is designed for the inner partial compartment, and the exterior is sealed with an integral sleeve. The radial dimensions of each compartment are φ50 mm. (1) The front and rear support claws are located at the two ends of the instrument, which are used to support the instrument to ensure that it is in the center of the pipe. Each end of the three claws is evenly distributed. One end of the claw is connected with the hinge of the instrument body, and the other end is connected by a crank slider. Using a spring return, the effective range of the claws is required to range from 85 mm to 120 mm. Two front support claws are designed with Hall switches and magnetic steel mounting holes for metering. (2) A battery cabin is required for loading the battery. The length of the cabin is 60 mm. It requires waterproof sealing, can withstand 0.5 MPa water pressure and is easy to disassemble when replacing the battery. (3) The instrument switch is arranged on the console. The mounting holes of the indicator and data cable plugs are required to be waterproof and sealed. (4) The circuit cabin is used to mount the circuit board with a length of 100 mm, leaving a threaded hole at the front and rear. (5) The sensor cabin has a length of 100 mm and is used to install gyroscopes and accelerometers. The mounting platform is an eccentric self-weighting platform that requires stable platform activity and does not flip with the instrument’s scrolling.

**Figure 5:**
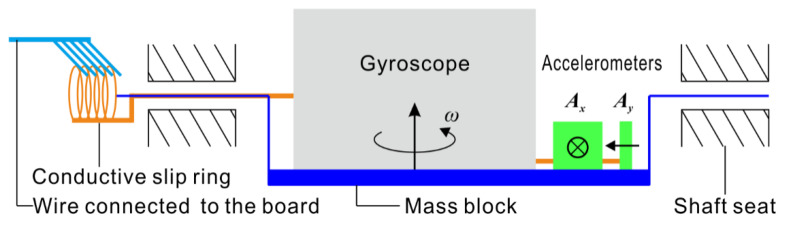
Schematic diagram of gravity stabilization platform.

To allow the measurement system to meet the semiplatform-semistrap condition constraints, it is essential to limit the pitch angle and roll angle range. First, the pitch angle is closely related to the direction of the pipeline. The maximum value is the maximum angle between the pipeline and the horizontal plane. When the pipeline is buried, its direction is limited to the horizontal direction. The pitch angle is generally small, and even the landslide has a large deformation, ensuring that the pitch angle does not flip, so the measurement of the pitch angle is already constrained when the measurement method is proposed, and the measurement can be directly performed. Second, the roll angle is caused by the rotation of the measuring system around the pipe axis when the system passes through the pipe. When the pipe has no limit groove to fix the degree of freedom of rotation of the measuring system around the z-axis, the rotation of the measuring system in the 360° range is unavoidable. To maintain the versatility of the pipeline, it is not recommended to make special modifications to the pipeline. Instead, the principle of platform inertial measurement is introduced onto the rolling shaft, and the eccentric weight of the platform is used to form a “tumbler” gravity platform, as shown in Figure 5. Thus, the range of the roll angle γ can be limited to the required range.

### 3.3. Measuring Circuit Design

**Figure 6:**
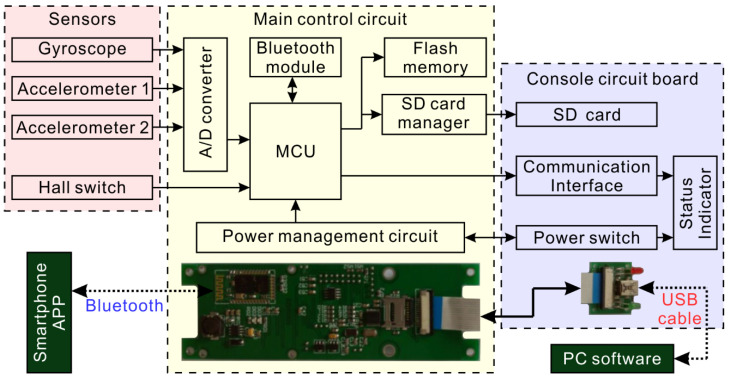
Block diagram of measuring system circuit.

The overall principle structure of the measurement circuit is shown in Figure 6. The measurement circuit includes functional circuits such as power management, sensor interfaces, data storage, and communication interfaces. The measuring circuit takes a single-chip microcomputer (MCU) as the core controller and controls the various functional circuits to work in order through the single-chip microcomputer program. To save MCU resources, the MCU in the circuit is only responsible for data acquisition, storage and communication and does not perform online operations on the data. Additionally, the circuit is equipped with a Bluetooth function module to remotely control the system’s operating status.

### 3.4. Pipeline Trajectory and Landslide Displacement Solution

**Figure 7:**
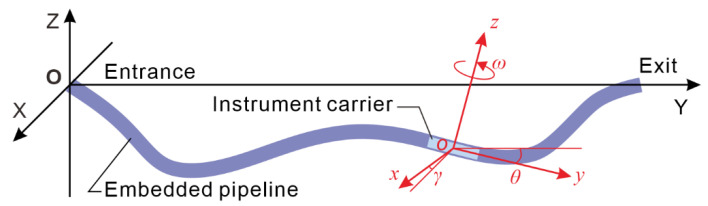
Reference coordinate system (OXYZ) and instrument coordinate system (*oxyz*).

Before calculating the pipeline trajectory, two coordinate systems are established (Figure 7): one is the reference coordinate system OXYZ, and OXYZ is fixed relative to the geodetic coordinate system and is a Cartesian coordinate system that satisfies the right-hand rule, wherein the Z-axis is perpendicular to the horizontal plane to the sky, and the *Y*-axis coincides with the pipe entrance and exit line alignment; the other is the instrument coordinate system oxyz, which is a Cartesian coordinate system that changes with the movement of the instrument, where the z-axis coincides with the sensitive axis of the gyroscope, and the x-axis and the y-axis coincide with the sensitive axes of the roll and pitch angle measurement accelerometers, respectively.

#### 3.4.1. Trajectory Attitude Solution

According to the measurement scheme and the measurement circuit, the measurement reference of the pitch θ and the roll γ (heeling angle $\gamma^{\prime }$) is gravitational acceleration, and the measurement reference of the yaw angular velocity ω is the angular motion inertia of the measurement system itself (the initial yaw is defined as $\psi_{0}$).

Regarded as the measurement reference for the pitch θ and the roll γ, the gravity vector is expressed as $b={\left(0,0,-g\right)}^{T}$. When the measurement system attitude is changed, the gravitational acceleration component measured on the two axes can be considered as the vector b generated by rotating the θ angle and the γ angle around the x-axis and the y-axis from the horizontal position. Use the Euler angle rotation matrix to describe the rotation process as:

(2) $$C_{O}^{1}=\left[\begin{array}{ccc} \cos\left(\theta \right) & 0 & \sin\left(\theta \right)\\ 0 & 1 & 0\\ -\sin\left(\theta \right) & 0 & \cos\left(\theta \right) \end{array}\right],C_{1}^{m}=\left[\begin{array}{ccc} 1 & 0 & 0\\ 0 & \cos\left(\gamma \right) & \sin\left(\gamma \right)\\ 0 & -\sin\left(\gamma \right) & \cos\left(\gamma \right) \end{array}\right],\;C_{O}^{m}=C_{1}^{m}\cdot C_{O}^{1}=\left[\begin{array}{ccc} 1 & 0 & 0\\ 0 & \cos\left(\gamma \right) & \sin\left(\gamma \right)\\ 0 & -\sin\left(\gamma \right) & \cos\left(\gamma \right) \end{array}\right]\left[\begin{array}{ccc} \cos\left(\theta \right) & 0 & \sin\left(\theta \right)\\ 0 & 1 & 0\\ -\sin\left(\theta \right) & 0 & \cos\left(\theta \right) \end{array}\right]\;=\left[\begin{array}{ccc} \cos\left(\theta \right) & 0 & \sin\left(\theta \right)\\ -\sin\left(\theta \right)\cdot \sin\left(\gamma \right) & \cos\left(\gamma \right) & \cos\left(\theta \right)\cdot \sin\left(\gamma \right)\\ -\sin\left(\theta \right)\cdot \cos\left(\gamma \right) & -\sin\left(\gamma \right) & \cos\left(\theta \right)\cdot \cos\left(\gamma \right) \end{array}\right]$$

where $C_{O}^{1}$ is a rotation matrix rotated by an angle of θ around the x-axis, $C_{1}^{m}$ is a rotation matrix rotated by an angle of γ around the y-axis, and $C_{O}^{m}$ is a comprehensive rotation matrix rotated by an angle of θ around the x-axis and then rotated by an angle of γ around the y-axis. (Reference coordinate system → measuring instrument coordinate system). Thus, the component of the gravity acceleration vector on the sensitive axis of the accelerometer of the instrument coordinate system is:

(3) $$A=\left[\begin{array}{c} A_{y}\\ A_{x}\\ A_{z} \end{array}\right]=C_{O}^{m}\left[\begin{array}{c} 0\\ 0\\ -g \end{array}\right]=\left[\begin{array}{c} -g\cdot \sin\left(\theta \right)\\ -g\cdot \cos\left(\theta \right)\cdot \sin\left(\gamma \right)\\ -g\cdot \cos\left(\theta \right)\cdot \cos\left(\gamma \right) \end{array}\right]$$

Note that the gravitational acceleration components produced by the rotation around the x- and y-axes are mapped on the y- and x-axes, respectively. Therefore:

(4) $$\{\begin{array}{l} \theta =\mathrm{acrsin}\left(-\frac{A_{y}}{g}\right)\\ \gamma =\mathrm{acrsin}\left(-\frac{A_{x}}{g\cdot \cos\left(\theta \right)}\right)\\ \alpha_{tilt}=\mathrm{acrcos}\left(-\frac{A_{z}}{g}\right)=\mathrm{acrcos}\left(\cos\left(\theta \right)\cdot \cos\left(\gamma \right)\right) \end{array}$$

where $\;\alpha_{tilt}$ is the inclination angle of the gyro platform relative to the horizontal plane.

Similarly, the relationship between the angular velocity $\omega_{m}$ measured in the instrument coordinate system and the yaw angular velocity $\omega_{Z}$ of the Z-axis in the reference coordinate system is:

(5) $$\omega_{m}=\left[\begin{array}{c} \omega_{m,x}\\ \omega_{m,y}\\ \omega_{m,z} \end{array}\right]=C_{O}^{m}\left[\begin{array}{c} 0\\ 0\\ \omega_{Z} \end{array}\right]=\left[\begin{array}{c} \omega_{Z}\cdot \sin\left(\theta \right)\\ \omega_{Z}\cdot \cos\left(\theta \right)\cdot \sin\left(\gamma \right)\\ \omega_{Z}\cdot \cos\left(\theta \right)\cdot \cos\left(\gamma \right) \end{array}\right]$$

Regarding the instrument coordinate system, only the gyroscope is placed on the z-axis, so only $\omega_{m,z}\;$ is valid in the above formula, which is expressed as:

(6) $$\omega_{m,z}=\omega_{Z}\cdot \cos\left(\theta \right)\cdot \cos\left(\gamma \right)$$

which is:

(7) $$\omega_{Z}=\frac{\omega_{m,z}}{\cos\left(\theta \right)\cdot \cos\left(\gamma \right)}$$

equivalent to:

(8) $$\omega_{Z}=\frac{\omega_{m,z}}{\cos\left(\alpha_{tilt}\right)}$$

Integrate $\omega_{Z}\;$ and find the yaw of the instrument projected on the horizontal plane as:

(9) $$\psi =\psi_{0}+\int^{\;}\omega_{z}\cdot dt$$

At this point, the instrument’s attitude (θ,γ,ψ) has been completely determined.

#### 3.4.2. Dynamic Correction of Attitude Angle

The measurement process of the instrument is a dynamic process. During the process of attitude angle derivation, the influence of the dynamic process on the measurement result is ignored. The neglected elements include the effect of the y-axis acceleration $a_{y}$ on the pitch θ and the effect of the x-axis and y-axis angular velocities $\omega_{x}$ and $\omega_{y}$ on $\omega_{Z}$.

##### Effect of the y-axis Acceleration on the Pitch

During the measurement process, there is a line motion on the y-axis, and $v_{y}$ cannot be guaranteed to move at a constant speed. Therefore, $a_{y}$ is not 0 and will be superimposed on the accelerometer-sensitive axis that measures the pitch angle. The result is:

(10) $$A_{y}=-g\cdot \sin\left(\theta \right)+a_{y}$$

The first-order derivative calculation for $a_{y}$ is:

(11) $$a_{y}=\frac{dv_{y}}{dt}$$

Then, the correction formula for θ is:

(12) $$\theta =\mathrm{acrsin}\left(-\frac{A_{y}-a_{y}}{g}\right)=\mathrm{acrsin}\left(-\frac{A_{y}-\frac{dv_{y}}{dt}}{g}\right)$$

##### Effect of the x-axis and y-axis Angular Velocities $\omega_{x}$ and $\omega_{y}$ on $\omega_{Z}$

During the dynamic measurement, the pitch θ and the roll γ are both varied, so the angular velocities $\omega_{x}$ and $\omega_{y}$ are not zero. Therefore, the angular velocity measurement reference vector should contain the other two axes, which are taken as ${\left(\omega_{X}\;,\;\omega_{Y}\;,\;\omega_{Z}\right)}^{T}$; then, Equation (5) is rewritten as:

(13) $$\omega_{m}=\left[\begin{array}{c} \omega_{m,x}\\ \omega_{m,y}\\ \omega_{m,z} \end{array}\right]=C_{O}^{m}\left[\begin{array}{c} \omega_{X}\\ \omega_{Y}\\ \omega_{Z} \end{array}\right]$$

To facilitate the calculation, the above matrix is rewritten into a transformation of $\omega_{m}\to \omega$, that is, the inverse transformation of the above matrix, as follows:

(14) $$\omega =\left[\begin{array}{c} \omega_{\text{X}}\\ \omega_{\text{Y}}\\ \omega_{\text{Z}} \end{array}\right]=C_{m}^{O}\left[\begin{array}{c} \omega_{m,x}\\ \omega_{m,y}\\ \omega_{m,z} \end{array}\right]=\left[\begin{array}{ccc} \cos\left(\theta \right) & 0 & -\sin\left(\theta \right)\\ 0 & 1 & 0\\ \sin\left(\theta \right) & 0 & \cos\left(\theta \right) \end{array}\right]\left[\begin{array}{ccc} 1 & 0 & 0\\ 0 & \cos\left(\gamma \right) & -\sin\left(\gamma \right)\\ 0 & \sin\left(\gamma \right) & \cos\left(\gamma \right) \end{array}\right]\left[\begin{array}{c} \omega_{m,x}\\ \omega_{m,y}\\ \omega_{m,z} \end{array}\right]\;=\left[\begin{array}{ccc} \cos\left(\theta \right) & -\sin\left(\theta \right)\cdot \sin\left(\gamma \right) & -\sin\left(\theta \right)\cdot \cos\left(\gamma \right)\\ 0 & \cos\left(\gamma \right) & -\sin\left(\gamma \right)\\ \sin\left(\theta \right) & \cos\left(\theta \right)\cdot \sin\left(\gamma \right) & \cos\left(\theta \right)\cdot \cos\left(\gamma \right) \end{array}\right]\left[\begin{array}{c} \omega_{m,x}\\ \omega_{m,y}\\ \omega_{m,z} \end{array}\right]\;=\left[\begin{array}{c} \cos\left(\theta \right)\cdot \omega_{m,x}-\sin\left(\theta \right)\cdot \sin\left(\gamma \right)\cdot \omega_{m,y}-\sin\left(\theta \right)\cdot \cos\left(\gamma \right)\cdot \omega_{m,z}\\ \cos\left(\gamma \right)\cdot \omega_{m,y}-\sin\left(\gamma \right)\cdot \omega_{m,z}\\ \sin\left(\theta \right)\cdot \omega_{m,x}+\cos\left(\theta \right)\cdot \sin\left(\gamma \right)\cdot \omega_{m,y}+\cos\left(\theta \right)\cdot \cos\left(\gamma \right)\cdot \omega_{m,z} \end{array}\right]$$

where $\omega_{m,\;x}$ and $\omega_{m,\;y}$ are the first-order derivatives of θ and γ, respectively:

(15) $$\{\begin{array}{c} \omega_{m,x}=\frac{d\theta }{dt}\\ \omega_{m,y}=\frac{d\gamma }{dt} \end{array}$$

then, the corrected $\omega_{Z}$ equation is:

(16) $$\omega_{Z}=\sin\left(\theta \right)\cdot \omega_{m,x}+\cos\left(\theta \right)\cdot \sin\left(\gamma \right)\cdot \omega_{m,y}+\cos\left(\theta \right)\cdot \cos\left(\gamma \right)\cdot \omega_{m,z}\;=\sin\left(\theta \right)\cdot \frac{d\gamma }{dt}+\cos\left(\theta \right)\cdot \sin\left(\gamma \right)\cdot \frac{d\theta }{dt}+\cos\left(\theta \right)\cdot \cos\left(\gamma \right)\cdot \omega_{m,z}$$

#### 3.4.3. Pipeline Trajectory Curve Calculation

The zero point of the yaw coincides with the *y*-axis, the counterclockwise direction is positive, and the clockwise direction is negative.

**Figure 8:**
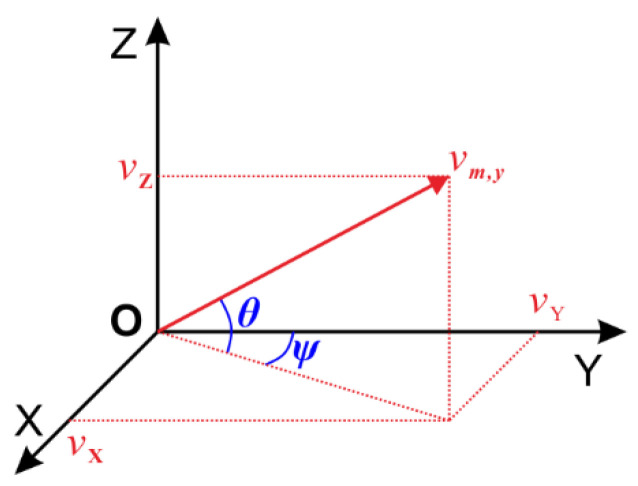
Geometric relationship between $v_{O}$ and $v_{m,y}$.

The linear velocity $v_{m,y}$ measured by the y-axis of the instrument coordinate system oxyz is sequentially rotated by θ and Ψ to the reference coordinate system OXYZ, and the velocity component of the instrument on the X-, Y- and Z-axes is obtained (as shown in Figure 8). The transformation process is:

(17) $$C_{m}^{1}=\left[\begin{array}{ccc} 1 & 0 & 0\\ 0 & \cos\left(\theta \right) & -\sin\left(\theta \right)\\ 0 & \sin\left(\theta \right) & \cos\left(\theta \right) \end{array}\right],C_{1}^{O}=\left[\begin{array}{ccc} \cos\left(\psi \right) & -\sin\left(\psi \right) & 0\\ \sin\left(\psi \right) & \cos\left(\psi \right) & 0\\ 0 & 0 & 1 \end{array}\right],\;C_{m}^{O}=C_{1}^{O}\cdot C_{m}^{1}=\left[\begin{array}{ccc} \cos\left(\psi \right) & -\sin\left(\psi \right) & 0\\ \sin\left(\psi \right) & \cos\left(\psi \right) & 0\\ 0 & 0 & 1 \end{array}\right]\left[\begin{array}{ccc} 1 & 0 & 0\\ 0 & \cos\left(\theta \right) & -\sin\left(\theta \right)\\ 0 & \sin\left(\theta \right) & \cos\left(\theta \right) \end{array}\right]\;=\left[\begin{array}{ccc} \cos\left(\psi \right) & -\cos\left(\theta \right)\cdot \sin\left(\psi \right) & \sin\left(\theta \right)\cdot \sin\left(\psi \right)\\ \sin\left(\psi \right) & \cos\left(\psi \right)\cdot \cos\left(\theta \right) & -\sin\left(\theta \right)\cdot \cos\left(\psi \right)\\ 0 & \sin\left(\theta \right) & \cos\left(\theta \right) \end{array}\right]$$

(18) $$v_{O}=\left[\begin{array}{c} v_{X}\\ v_{Y}\\ v_{Z} \end{array}\right]=C_{m}^{O}\left[\begin{array}{c} 0\\ v_{m,y}\\ 0 \end{array}\right]=\left[\begin{array}{c} -\cos\left(\theta \right)\cdot \sin\left(\psi \right)\\ \cos\left(\psi \right)\cdot \cos\left(\theta \right)\\ \sin\left(\theta \right) \end{array}\right]\cdot v_{m,y}$$

Through integrating $v_{O}$, the motion track of the instrument in the reference coordinate system OXYZ can be obtained, that is, the trajectory curve of the pipeline $S_{O}$:

(19) $$S_{O}=\left[\begin{array}{c} S_{X}\\ S_{Y}\\ S_{Z} \end{array}\right]=\left[\begin{array}{c} \int^{\;}v_{X}\cdot dt\\ \int^{\;}v_{Y}\cdot dt\\ \int^{\;}v_{Z}\cdot dt \end{array}\right]$$

#### 3.4.4. Landslide Displacement Calculation

Using the Y-axis as the reference datum, the two trajectory curves are subtracted to obtain the landslide displacement distribution D along the Y-axis.

$$D=\left[\begin{array}{c} D_{X}\\ D_{Y}\\ D_{Z} \end{array}\right]=\left[\begin{array}{c} S_{X,n}-S_{X,0}\\ S_{Y,n}\left(or\;S_{Y,0}\right)\\ S_{Z,n}-S_{Z,0} \end{array}\right]$$

where $S_{O,0}$ is the initial trajectory of the pipe, $S_{O,n}$ is the nth measured pipe trajectory, $D_{X}$ and $D_{Z}$ represent the horizontal displacement and vertical displacement of the landslide, respectively, and $D_{Y}$ represents the distribution position of the displacement on the Y-axis line.

## 4. Test Results and Application

### 4.1. Test Results

**Figure 9:**
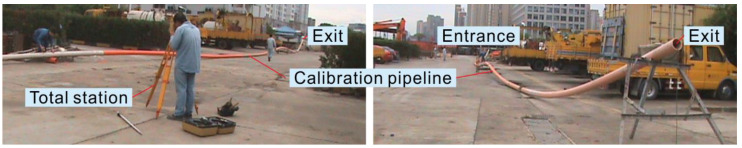
Instrument calibration test site.

**Figure 10:**
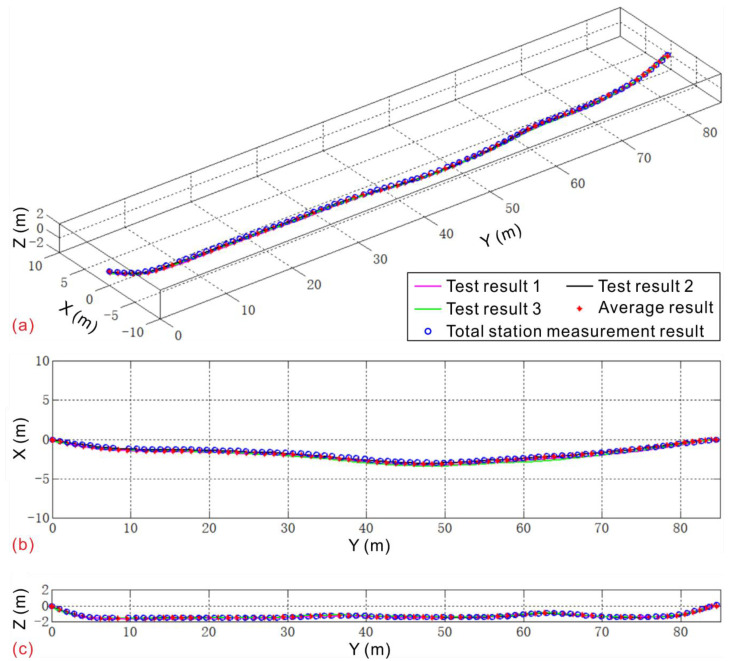
Calibration test results: (**a**) Axonometric view; (**b**) X–Y plane view; (**c**) Y–Z plane view.

Referring to the embedding of a deformed coupled pipe in the landslide body, a certain bending form of the pipeline is arranged on the ground, and the shape is measured by the total station instrument, as shown in Figure 9. The total station measurement trace curve is used as a reference to compare with the measurement results of the pipeline mapping system developed in this paper. The results showed that the random error of the pipeline mapping system can reach approximately 12 cm/100 m (max error/survey line span) in a single measurement, and it cannot meet the measurement requirements of the landslide displacement. However, by taking the average of multiple measurements in the same measurement cycle, the accuracy of the trajectory will increase significantly, improving to 3 cm/100 m (Figure 10).

### 4.2. Application

**Figure 11:**
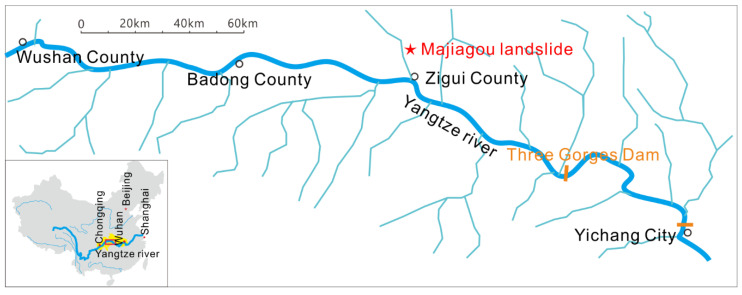
Majiagou landslide test site location.

**Figure 12:**
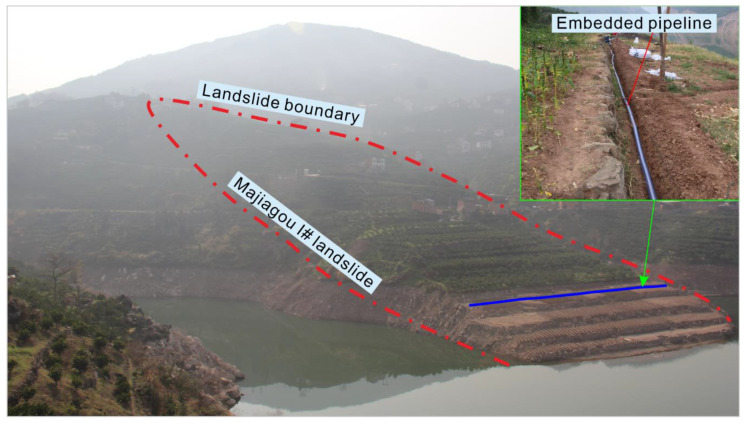
Majiagou I# landslide test site.

Verification application of the instrument was carried out in the Majiagou I# landslide. The Majiagou landslide is situated in Guizhou town, Zigui county, Hubei Province (Figure 11). It is a typical landslide affected by reservoir drawdown and rainfall in the Three Gorges reservoir area. The pipeline was laid out on the 170 m elevation terrace on the Majiagou landslide (Figure 12) to test and verify the pipeline mapping system developed in this paper. It should be noted that the buried pipeline is a spiral steel wire hose with strong radial pressure resistance and axial flexural deformation ability, which not only ensures patency inside the pipeline but also ensures deformation coupling coordination.

**Figure 13:**
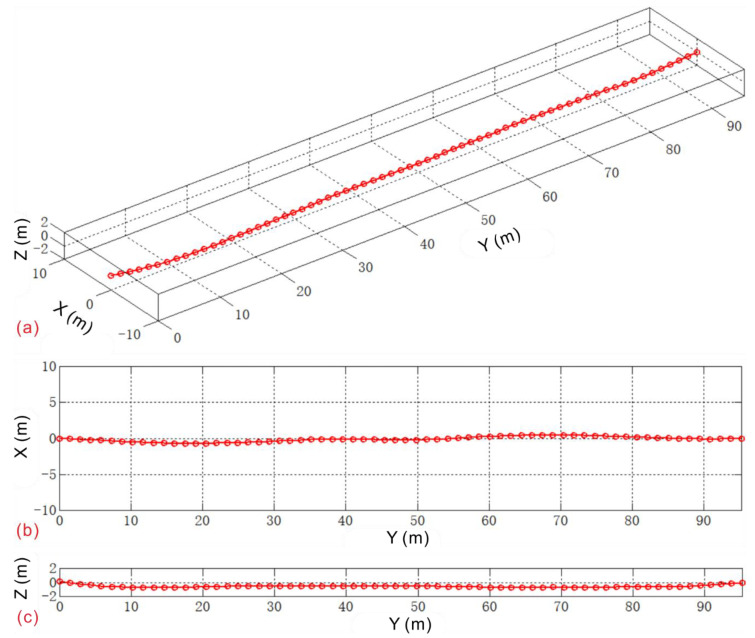
Initial pipeline trajectory curve of field test: (**a**) Axonometric view; (**b**) X–Y plane view; (**c**) Y–Z plane view.

The trajectory of the buried pipeline is measured by a lateral tracking plotter. The average trajectory curve obtained after three measurements is shown in Figure 13, and the length along the *y*-axis is 95.20 m. Concurrently, the initial absolute latitude and longitude coordinates of the inlet and outlet of the pipeline are measured by GPS. This is the initial trajectory of the pipe, which will be the zero point of the landslide displacement, and the deformed pipe trajectory will be acquired at regular intervals in the future.

## 5. Discussion

**Figure 14:**
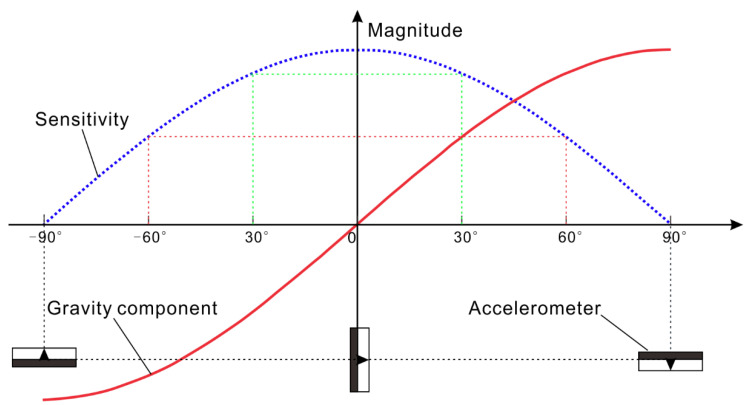
Sensitivity versus angle of accelerometer.

This pipeline trajectory is mainly designed for the horizontal distribution measurement of landslide displacement and is not recommended for the measurement of vertical distribution displacement. Considering the measurement principle alone, it is only necessary to avoid the elevation angle and the rolling angle of 90° in the instrument measurement. During the actual measurement, when the pitch angle and the roll angle are close to 90°, although the system can operate, the sensitivity of the sensor will decrease as the angle increases. According to the principle of measuring the angle of the accelerometer, the output of the accelerometer is the sine of the inclination angle, and the first derivative (also the sensitivity of the accelerometer as the angle sensor) is the cosine (Figure 14). Assuming that the output sensitivity of the sensor cannot be less than 0.3 times that of the equilibrium position, the result calculated by acos(0.3) ≈ 72.5° is that the pitch angle and the roll angle should not exceed 72.5°. Therefore, it is recommended that the coupling trajectory for this pipe trajectory is ±72.5°, and the measurement of the pipe trajectory at a larger inclination will be better with a borehole inclinometer.

Limited by the basic principle, the trajectory of the coupled pipe cannot reflect the lateral deformation of the landslide on the Y-axis, which should be considered in its application. Indeed, the displacement of the landslide is mainly composed of the horizontal displacement on the X-axis and the vertical displacement on the Z-axis, and the lateral displacement on the Y-axis is generally small and even negligible. Regarding special cases, if needed, this mapping system can be used together with the borehole inclination method to obtain the three-dimensional deformation distribution of the landslide.

The error mainly comes from the systematic error of the instrument, which is limited by the sensor model, hardware circuit, mechanical structure, machining, signal processing method, etc. To fundamentally reduce these errors, it is necessary to upgrade the instrument, including hardware and software. However, under the current instrument conditions, the method of obtaining the mean after multiple measurements can basically meet the measurement requirements of landslide displacement [23].

## 6. Conclusions

Here, a novel displacement measurement method was proposed. Since the method introduces a flexible pipeline coupled with landslide deformation and an inertial-measurement-based pipeline trajectory measuring instrument, it is important to introduce the instrument and measurement method as follows:

(1) Combination of sensor. Considering the engineering geological conditions, contact measurements were applied to measure the axial linear velocity of the pipeline, a single-axis gyro was adopted to measure the azimuth, and gravitational acceleration was selected as the reference physical variable of the roll angle and pitch angle. Therefore, the final combination of sensors was presented as a “single-axis gyro + two accelerometers + external roller coded program” and made up a simplified semiplatform-semistrap inertial measurement model.
(2) Configuration of mechanical structure. The instrument was enclosed in a modular cabin. The cabin was mainly comprised of the sensor compartment, circuit compartment, battery compartment, and console, and all were sealed in the cylinder sleeve. Additionally, two sets of support claws formed by six uniformly distributed rollers were assembled at both ends of the instrument, and a two-phase electromagnetic encoder was assembled on the roller for velocity measurement.
(3) Calculation of displacement distribution. The attitude angle, e.g., pitch angle, roll angle and azimuth angle at each measurement moment, was calculated by the monitoring results of the two-axis accelerometer and one-axis gyro. Then, according to an Eulerian transformation with regard to the attitude angle, the axial linear velocity was projected to a reference frame. Finally, the pipeline trajectory was calculated by the integration of three-axes-projected linear velocity, and the difference of trajectory monitored at each measurement moment was considered as the displacement distribution of a landslide. It is stressed that the accumulation of axial linear acceleration will affect the precision of the pitch angle when unavoidably added to the pitch angle accelerometer, which, however, should be removed in the calculation.
(4) Verification and application. Compared with the results of the total station and trajectory measurements applied in a simulating pipeline, the instrument exhibited a high precision of 3 cm/100 m in multiple singly periodic measurements, which meets the displacement measurement requirement of landslides with medium precision. Conversely, the instrument was successfully applied to the deformation monitoring of the Majiagou I# landslide when fully considering the engineering geological conditions.

## Figures and Tables

**Table 1:** Optional physical parameters of measurement.

Type of Movement	Axis	Physical Quantity	Measuring Device	Limiting Condition	Selection
Line motion	y	Linear acceleration	Accelerometer	None	
		Linear velocity	Encoder	Contact measurement	√
Angular rotation	x	Angular velocity	Gyroscope	None	
		Angle	Gravity accelerometer	Cannot be 90°	*√*
	y	Angular velocity	Gyroscope	None	
		Angle	Gravity accelerometer	Cannot be 90°	√
	z	Angular velocity	Gyroscope	Cannot be 90°	√
